# Betel Quid Health Risks of Insulin Resistance Diseases in Poor Young South Asian Native and Immigrant Populations

**DOI:** 10.3390/ijerph17186690

**Published:** 2020-09-14

**Authors:** Suzanne M. de la Monte, Natalia Moriel, Amy Lin, Nada Abdullah Tanoukhy, Camille Homans, Gina Gallucci, Ming Tong, Ayumi Saito

**Affiliations:** 1 Department of Pathology and Laboratory Medicine, Providence VA Medical Center, Providence, RI 02808, USA; 2 Women & Infants Hospital of Rhode Island, Providence, RI 02808, USA; 3 Alpert Medical School, Brown University, Providence, RI 02808, USA; 4 Departments of Medicine, Rhode Island Hospital, Providence, RI 02808, USA; Gina_Gallucci@URI.edu (G.G.); Mtong216@gmail.com (M.T.); 5 Neurology, Neurosurgery and Neuropathology, Rhode Island Hospital, Alpert Medical School of Brown University, Providence, RI 02903, USA; 6 Department of Molecular Pharmacology and Physiology at Brown University, Providence, RI 02912, USA; natalia_moriel@alumni.brown.edu (N.M.); amy_lin@alumni.brown.edu (A.L.); nada_abdullah@alumni.brown.edu (N.A.T.); 7 Department of Neuroscience, Brown University, Providence, RI 02912, USA; camilla_homans@brown.edu; 8 Department of Epidemiology in the School of Public Health, Brown University, Providence, RI 02912, USA; Ayumi_Saito@alumni.Brown.edu

**Keywords:** betel quid, arecoline, diabetes, dementia, tobacco, nitrosamine

## Abstract

Betel quid, traditionally prepared with areca nut, betel leaf, and slaked lime, has been consumed for thousands of years, mainly in the form of chewing. Originally used for cultural, medicinal, and ceremonial purposes mainly in South Asian countries, its use has recently spread across the globe due to its psychoactive, euphoric, and aphrodisiac properties. Now it is widely used as a social lubricant and source of financial profit. Unfortunately, the profit motive has led to high rates of habitual consumption with eventual conversion to addiction among young girls and boys. Moreover, the worrisome practice of including tobacco in quid preparations has grown, particularly among pregnant women. Major health concerns include increased rates of malignancy, oral pathology, and cardiovascular, hepatic, fertility, metabolic, and neuropsychiatric disorders. Metabolic disorders and insulin resistance disease states such as type 2 diabetes, obesity, and metabolic syndrome contribute to cognitive decline and neurodegeneration. Mechanistically, the constituents of areca nut/betel quid are metabolized to N-nitroso compounds, i.e., nitrosamines, which are carcinogenic at high doses and cause insulin resistance following chronic low-level exposures. From an epidemiological perspective, the rising tide of insulin resistance diseases including obesity, diabetes, and dementias that now disproportionately burden poor countries has been propagated by rapid commercialization and enhanced access to betel quid. Public health measures are needed to impose socially and ethically responsible barriers to yet another cause of global health disparity.

## 1. Introduction

Epidemiological trends reflecting progressively increased prevalence rates of insulin resistance diseases including diabetes mellitus, non-alcoholic fatty liver disease, and Alzheimer’s disease (AD)-type dementia in Asian (primarily Southern and South Eastern) countries are not readily explained since, until relatively recent times, they have been spared of preservative-laden highly processed Western foods and attendant morbid obesity [1]. On the other hand, the growing consumption of betel quid, particularly by adolescents and young people, is noteworthy because one of its main constituents, areca nut, contains alkaloids that get metabolized to nitrosamine compounds. Experimentally, low-dose nitrosamine exposures cause systemic insulin resistance diseases, cognitive impairment, and AD-type neurodegeneration [2,3]. In addition, the popular inclusion of tobacco in betel quid preparations could exacerbate the long-term metabolic and neurocognitive adverse effects of areca nut consumption in young people, as has been demonstrated previously in experimental models of low-dose tobacco nitrosamine exposures. This review was inspired by the need to assess the potential role of increased chronic consumption of betel quid, together with the co-consumption of tobacco, as sources of soaring nitrosamine exposures and driving forces for the rising rates of insulin resistance diseases including neurodegeneration in Southeast Asian populations.

## 2. Materials and Methods

This article was generated by a systematic review of primary and summary historical, social, economic, demographic, chemical, epidemiological, and clinical literature pertaining to areca nut/betel quid uses and consumption from 1919 to 2019. English language databases searched included PubMed, Google Scholar, Scopus, and government reports. Keywords and pairings included: areca nut or betel quid with consumption; exposure; youth; cultural practices; women; tobacco; metabolic; insulin; central nervous system; smoking; nitrosamine; diabetes; cognitive impairment; nicotine; arecoline; Asia; economics; preparation; metabolism; addiction; health; school age. Exclusion terms were as follows: malignancy; experimental animal model; signal transduction; aging; immunology; molecular biology; modern medicinal use; and genetics. Unfortunately, due to socioeconomic limitations and lack of adequate resources, most of the primary data were generated by observational, retrospective, and cross-sectional designs in selected regions or countries. However, to optimize robustness of this review, emphasis was placed on reproducible large-scale studies.

## 3. Areca Nut/Betel Quid—Historical Perspectives

Areca nut is the fruit of the *Areca catechu* tree, and for centuries it has been consumed by chewing, particularly in preparations known as “betel quid”. Betel quid preparation and chewing can be dated back to 10,000 BCE [4,5]. Descriptions of areca nut uses and effects across many South Asian countries were provided by Herodotus (340 BCE), Gupta period literature (499 BCE) [5,6,7], and Marco Polo’s 13th-century travel journals [8]. However, the habit of consuming areca nut may have originated in the Penang province of Malaysia, as the name translates directly to “areca nut” [9], or the Philippines which supports diverse wild forms of areca nut [6,10].

## 4. Betel Quid Preparations

The earliest and simplest preparations of betel quid required just three ingredients: crushed areca nut, slaked lime, and a betel leaf. The *Areca catechu* palm tree requires little care, has a lifespan of 60 to 100 years, and can produce up to 800 areca nuts per year. After removing its outer hard shell, the ripe or unripe areca nut can be chewed raw, boiled, sun-dried, benzoin-smoked, or salted and dried [5,11,12]. Slaked lime is prepared from available sources of calcium hydroxide such as limestone chalk, mollusks, or burnt coral. Betel leaves are from the *Piper betle* plant (vine). All ingredients needed to prepare betel quid can be cultivated or readily obtained in Thailand, India, Bangladesh, China, the Pacific Islands, Taiwan, and Vietnam [5].

Betel quid is prepared by first folding the betel leaf into a small pocket that can secure the other ingredients through several cycles of chewing and spitting. Crushed areca nut is mixed with slated lime as a paste and inserted into the betel leaf pocket [13]. Chewing and mixing the assembled betel quid with saliva yields a reddish liquid that is expectorated, retaining the betel quid for additional cycles of chewing and spitting over several hours. Traditional preparations have an astringent flavor, but later practices led to the inclusion of garnishments such as cardamom, ginger, anise, cloves, coconut, camphor, nutmeg, or tobacco [14]. Betel quid consumption is habit forming and often addictive due to the nicotine-like properties of arecoline, a major active component that produces sensations of alertness, euphoria, tachycardia, and vigor [15].

Over 600 million people worldwide regularly consume betel quid, making it the 4th most commonly used or abused psychoactive stimulant [12,16] after alcohol, caffeine, and tobacco [9,17]. Data compiled by the Food and Agricultural Organization of the United Nations (FAO) were tabulated to compare areca nut production by country (Table 1), geographical region (Table 2), and socioeconomic conditions (Table 3) http://www.fao.org/faostat/en/#rankings/countries_by_commodity. Table 1 shows areca nut production in 1990, 2000, 2010, and 2014 in the world and in 12 countries, along with their percentages of world production and the percentage changes in production over time. India has consistently been the highest producer of areca nut, followed by China/Taiwan. However, in contrast to India where areca nut production increased continuously, the levels in China/Taiwan peaked in 2000 and declined in 2010 and 2014 (Table 1). Of further note is that in Myanmar, Bangladesh, Thailand, Sri Lanka, and Bhutan, each of which accounted for less than 7 percent of the world’s production of areca nut in 1990, rapid growth in demand was reflected by their largest overall percentage increases in production through 2014. Areca nut production in Southern followed by South Eastern Asian countries peaked in 2010, but production in 2014 was still higher than that in 1990 and 2000 (Table 2). The FAO data also revealed that low-income food-deficit countries, net food importing countries, and the least developed countries had the most abundant and progressively increased areca nut production (Table 3) (http://www.fao.org/faostat/en/#data). Although all age groups were affected, the greatest impact of increased areca nut availability/production was in youths from socially and economically disadvantaged populations [12].

## 5. Cultural and Medicinal Uses of Areca Nut and Betel Quid

Due to its euphoric, psychoactive, and aphrodisiac effects, the practice of using betel quid at social gatherings and religious ceremonies and for wedding gifts has persisted for centuries to the present time [5,18]. Betel quid has been incorporated into the pharmacopeia of traditional (Ayurvedic) medicine for treating constipation, anxiety, inflammation and bloating, lost libido [14], depression, neuroinflammation, and pain [15,19,20,21,22]. However, of particular interest are the claims that betel quid enhances memory and cognition [19] vis-à-vis its growing use in young people including children, adolescents, and pregnant women, and high rates of dependency and addiction [13,23,24].

Arecoline has been shown to transiently modulate cognitive functions by improving focus, arousal, and learning [19,25] due to its agonistic effects on the muscarinic acetylcholine receptor, which is targeted in the treatment of early Alzheimer’s disease [19,26,27,28,29,30,31,32,33]. In their study, Ho, et al. demonstrated that betel quid chewing significantly reduced object masking via enhanced attentional processing [25]. On the other hand, in a more structured study that included 72 participants, Chiu et al. showed significant impairments in spatial short-term memory among habitual/dependent chewers compared with non-chewers, whereas visual or verbal short-term memory were unaffected [34]. Furthermore, in a limited study of 12 subjects, strong impairments in perception with slowed processing speed and compromised ability to think despite increased arousal, were associated with altered electroencephalographic activity [35]. The takeaway message is that while short-term exposures tend to produce positive stimulatory and medicinal effects, chronic betel quid consumption can adversely affect brain function by impairing cognition during critical phases of social, cultural, and educational maturation in young people.

## 6. Trends in Combining Areca Nut with Tobacco in Betel Quid

The substitution of tobacco for betel leaves in quid preparations contributes to dependence and addiction [36]. Case–control [37,38] and observational [39,40] studies in Southeast Asian and Pacific Island countries such as Cambodia, Indonesia, Thailand, Guam, and Malaysia revealed that women consume smokeless tobacco because society disapproves of their smoking [41]. For example, in India, over 90% of tobacco use by women occurs via chewing [39,42], and quid is the most popular preparation [43,44].

## 7. Betel Quid Economics 

Historically, *Areca catechu* palm trees were cultivated to meet individual and community needs [45], but over the past several decades, growing demand caused *Areca catechu* palm tree farming to be converted into a cash crop. Data from the Food and Agriculture Organization revealed substantial growth in areca nut production and profits led by India, followed by Indonesia, China, Myanmar, and Bangladesh [46] (Table 1 and Table 2). Several districts in India including Karnataka, Kerala, and Assam account for nearly 83% of the national production of *Areca catechu*, and millions throughout India are economically dependent upon the areca nut industry for subsistence [47]. Attempts to curtail problems of abuse and dependency through regulation have met resistance due to the profound adverse economic effects they would have on poor nations [48] (Table 3). 

Betel quid is one of the fastest-growing multi-million-dollar industries in Southeast Asian markets due to readily available non-perishable products, i.e., pan masala and gutka, and effective targeting of younger, poorly educated, and economically disadvantaged males and females [49,50,51,52,53,54]. Within a few decades of the initial marketing in the 1960s, annual growth of the gutka and pan masala industries increased by 25% to 30% [13,38]. Substantially lower taxes on pan masala versus that on cigarettes enable the products to be sold at affordable prices to teens and homeless or socioeconomically disadvantaged children [55].

Successful marketing to minors is best illustrated by examples provided in cross-sectional research. A 1994 study showed that 42% of boys and 13% of girls between ages 5 and 20 years consumed betel quid with tobacco [38]. Another investigation in the Maldives showed that 79% of children, 5–15 years old, had already acquired betel quid chewing habits [56]. In Sriperambudur Taluk interviews and focus group discussions, areca nut chewers 10 years of age and older revealed that people younger than 30 years tended to chew areca nut commercial products for recreation or in response to peer pressure whereas older people (30 to 50 years) chewed areca nut to alleviate boredom or stress from family problems [57]. In another study of 1162 Kaohsiung residents, 15 years and older, areca nut chewing was reported in 13.3%, mostly male, but the age of onset was younger (teen years), and consumption rates increased over time [58]. A major adverse effect of initiating areca nut consumption at very young ages is that it sets the stage for later life dependency and addiction.

An important source of economic growth is the continued consumption of betel quid by immigrant populations. Over the past several decades, the betel quid market has globalized, expanding to North American and European nations, largely due to immigration with transfer of cultural habits. In New York City, a questionnaire-based community survey of 138 first-generation Bangladeshi and Indian-Gujarati immigrants revealed that approximately 30 percent reported ever-regular use of gutka or paan [59]. Another survey of Bangladeshi women living in an immigrant community within the U.K. revealed up to 95% prevalence of habitual betel quid chewing, with 62% consuming tobacco with the quid [60]. In a later study, the finding that there were no differences in paan usage between first- and second-generation Bangladeshi women indicates that the habit can persist across generations, sustaining demand [61].

## 8. Betel Quid: Expanded Use through Gateway Exposures, Marketing, and Sex

The gateway to early areca nut exposures and habitual use or addiction is often through close relatives and friends. As perhaps an extreme example, in Papua New Guinea, mothers reportedly give pre-chewed betel quid to their very young children [11]. More commonly, the exposures begin between elementary school and junior high school because children and adolescents are heavily targeted and at high risk for initiating product usage [55,62,63]. Correspondingly, in a study conducted in Taiwan via focus groups and face-to-face interviews, participants (n = 41) indicated that betel quid initiation usually occurs during childhood via cultural or social venues, to achieve feelings of high energy and euphoria, or to combat boredom [64]. In a further questionnaire-based study of adolescents in Taiwan, the authors observed that betel quid chewing behavior was related to perceptions about the process, self-esteem, and locus of self-control [65], suggesting that youths have differential susceptibilities to gateway mediators of habitual areca nut chewing and therefore addiction. In a survey of 53,528 adolescent students between 12 and 18 years of age in Taiwan, betel-nut-only users, who were more likely to be male, reportedly had greater risks of anxiety, depression, and problems with thought and attention [66].

Among 1500 surveyed 1st to 8th grade children in Gujarat, India with a mean age of 11.8 years the prevalence of areca nut chewing was 33.3% for boys and 22.2% in girls, and the vast majority of the habits stemmed from exposures at 14 years of age or younger [67]. In a cross-sectional study of 1359 high school students, ages 14–18 in the Parsa district of Nepal, 30.4% were identified as habitual chewers, but the frequency of chewing areca nut was 82%, and for the commercial preparations, pan masala and gutka, the rates were 10.4% and 8.0%, respectively [68]. In a large community-based survey of Sriperambudur Taluk in rural India, early exposures to areca nut started between 13 and 15 years of age and were later followed by increased rates of smoking and alcohol consumption [57]. Similar problems have been documented in Micronesia and Pakistan. In a self-administered questionnaire-type survey of 309 high school children in Saipan, Micronesia, regular habitual chewing of areca nut was 63.4%, which is the highest level reported for this age group [24].

Either deliberate sophisticated consumer marketing or inadvertent targeting has resulted in disproportionately high rates of betel quid chewing habits among socioeconomically and educationally disadvantaged populations including children and adolescents. The availability of non-perishable, readily available forms of betel quid further increase the susceptibility of young people to engage in its consumption [13]. Among 160 school-attending children ages 4–16 in Karachi, Pakistan, 74% used areca nut, 35% consumed it daily, and rates of use increased with grade level [23]. Low socioeconomic status was correlated with addiction potential and the use of areca nut products containing tobacco [23]. In another cross-sectional survey of 2140 high school students in Karachi, Pakistan, betel quid usage was 42.6%, and higher rates of use correlated with easy accessibility outside school, parental education, and peer pressure [69]. Similar trends in areca nut chewing were identified in a stratified cluster random sample of 2442 junior high school students in Changhua, Taiwan [70], and the trends were higher in vocational and agricultural students compared with that among general school students [71]. Early (elementary school) exposures, peer pressure, and poor education were identified as important factors associated with habitual betel chewing in a cross-sectional study of Taichung County (Taiwan) junior high school students [72], corresponding with data obtained in Karachi, Pakistan [23].

Sex is an important factor driving betel quid consumption (http://articles.latimes.com/2009/jan/22/world/fg-betel-beauty22) and (https://www.metro.us/news/selling-betel-nut-with-sex/tmWicq---33KCBLjNAY7o; https://www.dailymail.co.uk/travel/travel_news/article-3244562/The-scantily-clad-girls-wait-neon-booths-sell-not-sex-NUTS-Inside-Taiwan-s-contro). For example, in Taiwan, betel nut girls run up to cars at drive-through services and allow their breasts to be fondled while handing over a packet of betel quid. For higher fees, the so-called betel nut beauties or binlang girls, attired in revealing clothes jump into the cars to perform oral sex. This type of marketing greatly expanded the industry in urban, suburban, and rural areas of Taiwan.

## 9. Adverse Health Effects of Chronic Betel Quid and Tobacco Exposures

Regular consumption of areca nut is habit forming and leads to dependency or addiction [73,74,75]. Long-term adverse health effects of habitual betel quit chewing include dark red staining and eventual blackening of teeth, periodontitis [76], submucosal fibrosis, and oral leukoplakia [38,77]. Additional long-term systemic illnesses include cardiovascular disorders associated with tachycardia, arrhythmias, and hypertension [78,79,80]; hepatic dysfunction; metabolic syndrome; erectile dysfunction; and infertility [81,82,83]. Central nervous system (CNS) consequences of betel quid dependency include deficits in spatial memory, pathophysiologic alterations in brain structure and resting activity [36], depression, and anxiety [84].

The impact of betel quid exposures during development can be devastating. The short-term psychoactive and physiological effects of arecoline in areca nut resemble those of nicotine, and both are addictive. Prenatal and early postnatal exposures to tobacco are known to cause low birth weight, sudden unexpected infant death, asthma, allergies, and neurodevelopmental deficits [38,85,86,87,88]. Similarly, in cross-sectional surveys of 310 pregnant woman in Papua New Guinea [89] and 1264 aboriginal women in Taiwan [90], betel quid chewing during pregnancy significantly impaired fetal development, increased risk of low birth weight, and led to neonatal withdrawal syndrome [89,91]. Although data from large cross-sectional surveys support the concept that areca nut/betel quid chewing during pregnancy poses risks to fetuses and endangers the quality of postnatal and childhood development, study-to-study findings are not universally concordant and growing evidence suggests roles for co-consumption of tobacco and/or alcohol during pregnancy [90,92,93]. In a retrospective cohort analysis of 1171 pregnant Palauan women who delivered between 2007 and 2013, consumption of betel quid with tobacco significantly increased risk for adverse birth outcomes including preterm delivery and low birth weight [94]. Therefore, a major concern is that in young reproductive-age women, consumption of “hidden tobacco” incorporated into betel quid has the potential to cause permanent harm to developing fetuses. Clearly, additional research is needed to address the true impact of these practices on maternal–fetal health.

## 10. Physiology, Chemistry, and Direct Actions of Areca Nut and Nicotine

Both areca nut and tobacco have short- and long-term effects on the body. The four main alkaloids present in areca nut are, arecoline, which comprises approximately 1% of the nut’s dry mass [95]; arecaidine, a prominent metabolite of arecoline; guvacoline, which is second in abundance after arecoline; and guvacine [81]. Arecaidine and guvacine are relatively minor components of the areca nut as their presence is often undetectable by mass spectrometry. Arecoline (C_8_H_13_NO_2_) (MW = 151.397), the dominant psychoactive component in areca nut, has 3 hydrogen bond acceptor sites enabling it to form two main metabolites: arecoline-N-Oxide via N-oxidation, and arecaidine via carboxylesterase-mediated hydrolyzation [96]. Arecaidine (C_7_H_11_NO_2_) (MW = 141.17) also has 3 main hydrogen bond acceptor sites as well as 1 hydrogen bond donor site. Guvacine (MW = 127.14 Da) is a secondary amino compound that acts as an alpha–beta unsaturated nicotinic carboxylic acid. Guvacoline (C_7_H_11_NO_2_) (MW = 141.17) is a derivative of guvacine and a beta-amino acid ester.

The short-term psychoactive effects of arecoline in areca nut and nicotine in tobacco, promote their habitual use, dependency, and eventual addiction due to their excitatory actions on the parasympathetic nervous system. The other major alkaloids in areca nut are arecaidine, guvacoline, and guvacine [97]. Arecoline’s stimulation of the parasympathetic nervous system is mediated by its agonistic effects on the M1, M2, and M3 muscarinic acetylcholine receptors [98,99]. Arecoline is also lipophilic and readily crosses the blood–brain barrier [38], enabling it to exert transient or sustained effects on the central nervous system (CNS). Experimentally, arecaidine was shown to act as a competitive inhibitor of gamma amino butyric acid (GABA) reuptake in mice, potentially accounting for the euphoria and behavioral changes associated with its use [100]. In animal models, arecoline increases dopamine transmission via mesocorticolimbic circuitry [36], which could be an important mediator of its habitual and dependent usage in young people.

## 11. Arecoline Metabolism, Pathophysiology, and Disease

Arecoline constitutes between 1% and 1.4% of the dry mass of areca nut [95]. Following oral consumption, arecoline can be detected in saliva, blood, urine, hair, and breast milk [26,101,102,103]. Arecoline is largely metabolized in the kidney and liver [104,105], although it encounters an initial metabolic bypass in the oral cavity, where it is incompletely metabolized by salivary enzymes [106]. Carboxylesterases hydrolyze arecoline and its main carboxylic acid metabolite, arecaidine [104]. Due to its nitrogen heterocyclic nature [107], further enzymatic breakdown of arecoline and arecaidine yields N-nitroso compounds, i.e., nitrosamines, including N-methylnipecotic acid, N-methylnipecotylglycine, and arecoline N-oxide [19,104].

Although the full spectrum of arecoline-derived nitrosamines and their downstream effects on the body are not known, the International Agency for Research on Cancer has classified areca nut as a Group 1 carcinogen [38]. Accordingly, chronic consumption, e.g., chewing, of betel quid prepared in the traditional manner, i.e., without tobacco, causes DNA damage [108] leading to oral squamous cell [38,109,110] and esophageal [38,54,111] carcinomas. Areca-nut-associated oral cancer has been documented in South Asian immigrant communities residing in Canada, Germany, or France [50,112]. Consequently, Canada and other countries have banned areca nut products. However, in the United States, pan masala and gutka can be acquired readily at local bars or via internet commerce as only interstate transportation is prohibited [38,76]. Presumably, the carcinogenic potential of areca nut could be reduced by increasing the efficiency of arecoline metabolism or lowering its content during preparation. Modest reductions in arecoline content from 1.4% to 1.35% or 1.29% can be achieved by sun drying or roasting, respectively. In contrast, arecoline content can be reduced to 0.7% by soaking the areca nuts in water, and down to 0.1% by boiling [38,113].

## 12. Nicotine Metabolism, Pathophysiology, and Disease

Although tobacco contains several alkaloids including nicotine, nornicotine, anabasine, anatabine, and myosmine, only nicotine, which comprises between 1% and 2% of the dry mass of unburned tobacco is responsible for its addictive properties [3]. Nicotine rapidly enters the circulation and distributes to various organs, including the liver, kidney, spleen, lung, and brain [38,114], and due to its lipophilic properties, nicotine crosses all membranes, including the skin and placenta [115] and penetrates the blood–brain barrier [114]. Its binding to nicotinic cholinergic receptors causes the release of neurotransmitters such as dopamine, norepinephrine, serotonin, and GABA, resulting in feelings of well-being, relaxation, calmness, alertness, and euphoria, and suppression of appetite, pain, and anxiety [116,117].

Nicotine is rapidly metabolized by cytochrome P450 (CYP) in the liver, lung, kidney, nasal mucosa, and brain [118]. Oxidation of the initial hydroxyl intermediate yields cotinine, which is more stable [119], but eventually gets further metabolized by glucuronidation and excreted in the urine. However, CYP2A6 can hydroxylate nicotine to form 2′-hydroxynicotine, which spontaneously forms an amino ketone (4-(methylamino)-1-(3-pyridyl)-1-butanone) intermediate, and subsequent nitrosation of the amino ketone yields tobacco procarcinogenic nitrosamines, including nitrosamino ketone (NNK), nitrosamino aldehyde (NNA), and N’-nitrosonornicotine (NNN), and procarcinogen, nitrosamine ketone (NNK) [120]. Among these, NNK is the most potent procarcinogenic and genotoxic agent. Its metabolites form DNA adducts that drive reactive-species-mediated DNA damage, unscheduled DNA synthesis, and chromosome aberrations [3,121,122,123]. Like arecoline, NNK has been classified as a Group 1 carcinogenic agent by the Working Group of the International Agency for Research on Cancer (IARC) [38]. Chronic exposure to tobacco leads to cancer development in many organs and tissues, but most notably in the oral cavity, lungs, liver, and pancreas [124,125,126,127].

Limited research suggests that the addition of tobacco to betel quid exacerbates the adverse health effects of either exposure alone. For example, chewers of areca nut with tobacco had higher levels of C-reactive protein, a marker of systemic inflammation compared with subjects who chewed only areca nut [128]. The severity of periodontal disease is worse in people who consume gutka than in those who chew betel quid without tobacco [129]. Moreover, the risk of developing oral cancer is higher among gutka users than that in chronic consumers of smokeless tobacco [130]. Unfortunately, the aggregate information extracted from earlier studies does not adequately distinguish the adverse health effects of areca nut + tobacco and the consequences of the individual exposures. Design flaws were mainly due to lack of awareness about the potential additive or synergistic adverse effects of areca nut + tobacco compared with those of either exposure alone [131,132,133]. Mechanistically, both the alkaloids in areca nut, including arecoline, and tobacco-specific nitrosamines can cause cellular injury, DNA damage, and oxidative stress and their effects could potentially be additive or synergistic.

## 13. The Rising Tide of Insulin Resistance Diseases and Cognitive Impairment in High Betel Quid Producing and Consuming Nations

**Figure 1:**
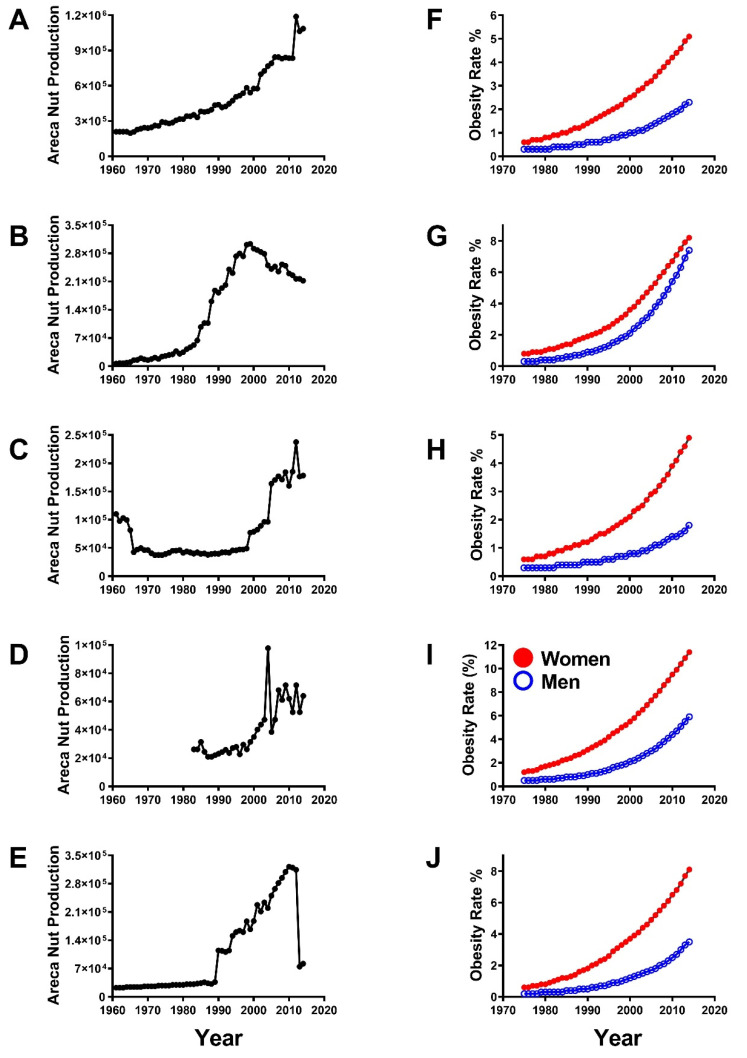
Comparisons of time-dependent increases in (**A**–**E**) areca nut production (gross production value constant 1000 1$) and (**F**–**J**) obesity rates among women (red) and men (blue) in (**A**,**F**) India, (**B**,**G**) China, (**C**,**H**) Bangladesh, (**D**,**I**) Thailand, and (**E**,**J**) Indonesia. Year over year data were extracted from the Food and Agricultural Organization of the United Nations website.

For centuries, the prevalence rates of obesity, type 2 diabetes mellitus, metabolic syndrome, and dementia were low in Southeast Asian populations. However, increased areca nut consumption has been associated with glucose intolerance or type 2 diabetes in both mice and humans [128,134,135,136,137,138,139,140,141,142,143,144,145,146]. Correspondingly, within the past several decades, the incidence and prevalence rates of insulin resistance diseases such as obesity have skyrocketed along with epidemics in areca nut production in betel quid consuming nations such as India, Bangladesh, China, Indonesia, and Thailand [147] (Figure 1). Links between increased areca nut consumption and higher incident rates of metabolic disorders such as diabetes mellitus were established in humans by demonstrating that betel quid usage increased waist size and weight in a British community of Southeast Asian immigrants [142]. Population-based studies in Taiwan revealed dose-dependent increases in central and general obesity [148], and higher prevalence rates of metabolic syndrome [146] and type 2 diabetes [149] in areca nut chewers. Under conditions of starvation including under-nutrition, areca nut has toxic effects on the endocrine system leading to thyroid dysfunction and metabolic stress [150]. Experimentally, areca nut exposures induced glucose intolerance in adult mice, as well as in the F1 and F2 offspring of betel-fed parents [135,136], reflecting transgenerational transmission of disease.

Insulin resistance in type 2 diabetes and obesity is a critical factor driving cognitive decline, especially in verbal memory, learning, and mental speed, suggesting that chronic areca nut consumption also could be associated with cognitive impairment [151,152,153]. Recent studies of addiction revealed morphological, functional, metabolic, and behavioral impairments in areca-nut-dependent users, including chronic deficits in spatial memory [154]. Likewise, independent and unrelated experimental findings have convincingly demonstrated causal relationships between chronic nitrosamine exposures and insulin resistance disease states including cognitive motor impairments associated with Alzheimer-type neurodegeneration [2,25,155,156,157,158,159,160]. Increased betel quid consumption correlates with greater availability of manufactured non-perishable forms as well as the inclusion of tobacco rather than betel leaves in the quid. Sadly, aggressive marketing, increased availability of processed and preserved products, and the lack of adequate education needed to make prudent health-related choices have sharply increased betel quid’s popularity among young adults, adolescents, and children from low socioeconomic backgrounds [13,38], in part due to the targeting of sales.

## 14. Mechanisms of Arecoline-Mediated Cellular and Tissue Injury

Arecoline-associated cellular and tissue injury and disease patterns largely correspond with its chemical and metabolite distributions. Toxic effects of arecoline metabolites have been detected in bone marrow cells, lymphocytes, neuronal cells, hepatocytes, myoblasts, and endothelial cells [161]. In some respects, the oral mucosa is partially protected from arecoline-induced oxidative injury due to the activation of salivary antioxidant mechanisms including glutathione release [162]. However, in cortical neurons, arecoline reduces glutathione levels and superoxide dismutase activity, suggesting that brain damage resulting from arecoline exposures is mediated in part by oxidative injury [163]. Correspondingly, several studies have demonstrated that treatment with agents that prevent or reduce oxidative stress, such N-acetyl-L-cysteine [164], Nicotinamide adenine dinucleotide phosphate (NAPDH) oxidase inhibitors, catalase [163], and vitamins C and E [165] can reduce the cytotoxic effects of arecoline.

Furthermore, there is evidence that apart from its direct effects, toxic metabolites of arecoline may have critical roles as mediators of cellular injury. Importantly, the generation of toxic metabolites may be driven by the inclusion of lime and nitrites in the betel quid preparations. Correspondingly, treatment of areca nut with lime increased the rate of arecoline hydrolysis to arecaidine [100]. In addition, synergistic toxicity resulted when human epithelial type 2 cells were exposed to arecoline plus sodium nitrite compared with that for either agent alone [166] due to the enhanced nitrosation of arecoline by nitrite. These findings suggest that foods and food preparations could potentially modulate the toxicity of betel quid.

## 15. Potential Dietary Measures to Reduce Adverse Effects of Chronic Betel Quid Consumption

Mastication of betel quid causes areca nut to be saturated with saliva and exposed to major salivary enzymes including amylases, lipases, and proteases, and an array of minor salivary enzymes such as cholinesterases. Arecoline metabolism in the oral cavity is partly achieved through the actions of amylase and cholinesterase, which function optimally at pHs ranging from 6.7 to 7.4. Increased salivary enzyme metabolism of arecoline could potentially limit the quantities that enter the gastrointestinal tract and systemic circulation and exert end-organ damage. Since various food substances can stimulate the secretion of saliva and salivary enzymes and also influence the pH of saliva, we hypothesize that dietary manipulation could potentially be used to reduce partial oral metabolism of arecoline and the attendant local accumulation of toxic metabolites that cause oral and esophageal cancers. We hypothesize that inefficient partial metabolism by salivary enzymes poses a greater health risk than bypassing the process to permit more complete metabolism in the liver. For example, leafy green spinach has a basic pH and is highly rich in nitrate [167]. The high content of nitrate increases nitrite concentrations in saliva [106], which in turn could lead to toxic nitrosation of arecoline [168]. Thus, it is unlikely that spinach consumption would protect against the toxic effects of arecoline.

Grapefruit, although highly acidic (pH 3), only transiently decreases the pH of saliva because soon after its ingestion, sodium bicarbonate is secreted by the salivary glands, increasing pH and diminishing enzymatic activity [169]. Blueberries contain polyphenols that have well-established health benefits. Moreover, blueberry tannins bind to proline-rich protein in saliva [170,171], increasing salivary prolines that could inhibit the nitrosation and metabolism of arecoline [172]. Turmeric, with its principal component curcumin, is abundantly available in Southeast Asian dishes and is well recognized for its therapeutic properties. However, turmeric lowers amylase levels in saliva [173] and, consequently, could inhibit the breakdown of arecoline. Anise/fennel is used as a breath freshener in Southeast Asia. Fennel and anise seeds both increase oral pH [174]. Elevation of salivary pH above 7.4 has the potential to reduce enzyme activity and decrease the metabolism of arecoline. Therefore, future studies should assess the protective effects of regular grapefruit, blueberry, turmeric, or anise/fennel seed consumption immediately before or after betel quid mastication as potential public health strategies that could be implemented easily in at-risk Southeast Asian populations.

## 16. Conclusions

This article reviews the historical, cultural, social, and economic perspectives on the growing global consumption of betel quid. Due to increased availability of processed and preserved betel quid, combined with commercialization, particularly in relation to sex and promise of “feel good states of mind,” exposures have shifted to highly vulnerable populations, particularly school-age boys and girls, pregnant women, and the poor. Unfortunately, a major side effect of habitual usage is addiction. Growing evidence indicates that toxic components of areca nut used to prepare quid promote a range of metabolic diseases including diabetes and metabolic syndrome, i.e., insulin resistance. These effects can be exacerbated by the popular addition of tobacco to the preparations. The authors hypothesize that nitrosamines present in areca nut and tobacco mediate these significant adverse health effects which, based on demographic trends, are now disproportionately impacting young vulnerable populations from poor nations. Although more research is needed, natural dietary interventions could aid in reducing the long-term toxic-metabolic effects of quid.

Despite significant health concerns related to addiction and chronic diseases and significant adverse impact from fetal through late adolescent and early adult development and maturation, policies to govern production, distribution, and sales of areca nut and betel quid have remained stagnant. Obstacles to progress include heavy lobbying from industry representatives together with the scarcity of objective, quantitative, and evidence-based clinical and experimental data showing detrimental effects of early life exposures to areca nut/betel quid, either alone or combined with tobacco.

## Figures and Tables

**Table 1:** Areca nut production by country, 1990–2014.

Territory	Production (mt)		Production (mt)		%Change	Production (mt)		%Change	Production (mt)		%Change	%Change
	1990	%World	2000	%World	1990–2000	2010	% World	2000–2010	2014	%World	2010–2014	1990–2014
World	868,124.0	100.00	1,271,098.8	100.00	46.42	1,889,697.0	100.00	48.67	1,928,986.5	100.00	2.08	122.2
India	416,734.4	48.00	576,613.6	45.36	38.36	835,216.1	44.20	44.85	1,086,829.3	56.34	30.13	160.8
China/Taiwan	194,109.1	22.36	291,757.8	22.95	50.31	230,185.9	12.18	−21.10	212,185.1	11.00	−7.82	9.3
Indonesia	113,884.7	13.12	187,710.4	14.77	64.83	322,030.0	17.04	71.56	82,123.8	4.26	−74.50	−27.9
Myanmar	56,385.8	6.50	68,313.0	5.37	21.15	206,183.1	10.91	201.82	207,772.2	10.77	0.77	268.5
Bangladesh	39,768.9	4.58	78,629.1	6.19	97.72	160,195.5	8.48	103.74	178,226.0	9.24	11.26	348.2
Thailand	24,323.3	2.80	34,946.3	2.75	43.67	62,160.3	3.29	77.87	64,073.9	3.32	3.08	163.4
Malaysia	6989.3	0.81	5766.1	0.45	−17.50	1135.8	0.06	−80.30	643.0	0.03	−43.38	−90.8
Sri Lanka	5241.9	0.60	15,997.9	1.26	205.19	52,209.7	2.76	226.35	73,347.0	3.80	40.49	1299.2
Bhutan	4368.3	0.50	5416.7	0.43	24.00	12,720.4	0.67	134.84	16,581.3	0.86	30.35	279.6
Nepal	4193.6	0.48	5766.1	0.45	37.50	7454.0	0.39	29.27	7002.4	0.36	−6.06	67.0
Kenya	186.1	0.02	157.3	0.01	−15.48	195.7	0.01	24.45	197.3	0.01	0.82	6.0
Maldives	28.0	0.00	24.5	0.00	−12.50	10.5	0.00	−57.14	5.2	0.00	−50.00	−81.3

Data corresponding to areca nut production by country from 1990 to 2014 were extracted from the Food and Agricultural Organization of the United Nations website. Total production data (gross production value constant 1000 1$) in 1990, 2000, 2010, and 2014 are shown for the world, 11 Asian countries, and 1 African nation. The calculated percentages of world production and percentage change in production during 1990–2000, 2000–2010, 2010–2014, and 1990–2014 are shown. The numbers in red font correspond to relative decline in Areca nut production within the specified intervals. Mt—metric tons

**Table 2:** Areca nut production by Asian region, 1990–2014.

	Production (mt)		Production (mt)		%Change	Production (mt)		%Change	Production (mt)		%Change	%Change
	1990	%World	2000	%World	1990–2000	2010	%World	2000–2010	2014	%World	2010–2014	1990–2014
World	868,124	100	1,271,099	100	46.42	1,889,697	100	48.67	1,928,987	100	2.08	122.2
Asia	867,938	99.98	1,270,942	99.99	46.43	1,889,501	99.99	48.67	1,928,789	99.99	2.08	122.2
Southern	472,246	54.40	682,448	53.69	44.51	1,067,806	56.51	56.47	1,361,991	70.61	27.55	188.4
South Eastern	201,583	23.22	296,736	23.34	47.20	591,509	31.30	99.34	354,613	18.38	−40.05	75.9
Eastern	194,109	22.36	291,758	22.95	50.31	230,186	12.18	−21.10	212,185	11.00	−7.82	9.3
Africa	186	0.02	157	0.01	−15.48	196	0.01	24.45	197	0.01	0.82	6.0

Data corresponding to areca nut production by Asian region and compared with Africa and the world from 1990 to 2014 were extracted from the Food and Agricultural Organization of the United Nations website. Total production data (gross production value constant 1000 1$) in 1990, 2000, 2010, and 2014 are shown for the world, Southern, South Eastern and Eastern Asian countries, and 1 African nation. The calculated percentages of world production and percentage change in production from 1990–2000, 2000–2010, 2010–2014, and 1990–2014 are shown. The numbers in red font correspond to relative declined in areca nut production within the specified intervals. Note virtually continuous growth in Asian regions and very low production in Africa.

**Table 3:** Areca nut production by Asian economy, 1990–2014.

	Production (mt)		Production (mt)		%Change	Production (mt)		%Change	Production (mt)		%Change	%Change
Economy	1990	% World	2000	% World	1990–2000	2010	% World	2000–2010	2014	% World	2010–2014	1990–2014
Low income	463,259	53.36	661,166	52.02	42.72	1,003,061	53.08	51.71	1,272,255	65.95	26.84	174.6
Food importer	112,083	12.91	174,305	13.71	55.51	438,969	23.23	151.84	483,131	25.05	10.06	331.0
Least developed	106,627	12.28	158,125	12.44	48.30	386,553	20.46	144.46	409,582	21.23	5.96	284.1
Land-locked	8096	0.93	11,183	0.88	38.12	20,174	1.07	80.41	23,584	1.22	16.90	191.3
Small island	28	0.00	24	0.00	−12.50	10	0.00	−57.14	5	0.00	−50.00	−81.3

Data corresponding to areca nut production by Asian economy from 1990 to 2014 were extracted from the Food and Agricultural Organization of the United Nations website. Low income = low income food deficit countries; Food importer = net food importing countries; Least developed = least developed countries; Land locked = land locked developing countries; Small island = small island of developing states. Total production data (gross production value constant 1000 1$) in 1990, 2000, 2010, and 2014 are shown for each economy. The calculated percentages of world production and percentage change in production from 1990–2000, 2000–2010, 2010–2014, and 1990–2014 are shown. The numbers in red font correspond to relative declined in Areca nut production within the specified intervals. Note virtually continuous growth in most economically challenged countries except for the small island developing states, which exhibited sharp declines in production.
